# C-Reactive Protein and Risk of Parkinson's Disease: A Systematic Review and Meta-Analysis

**DOI:** 10.3389/fneur.2019.00384

**Published:** 2019-04-17

**Authors:** Xiaohui Qiu, Yousheng Xiao, Jingjing Wu, Lu Gan, Yanning Huang, Jin Wang

**Affiliations:** Department of Neurology, The First Affiliated Hospital of Guangxi Medical University, Nanning, China

**Keywords:** Parkinson's disease, C-reactive protein, risk factor, meta-analysis, inflammation

## Abstract

**Background:** C-reactive protein (CRP) has been identified as a common inflammation-related cytokine. Although publications indicate that CRP is associated with the pathogenesis of neurological disorders and deemed to be a “risk factor” for Parkinson's disease (PD), the evidence exists still indefinitely. Here, we performed a systematic review with meta-analysis synthesizing all the eligible studies on serum, plasma, and cerebrospinal fluid (CSF) CRP levels and PD risk to investigate the potential relevance.

**Methods:** A systematical search up to October 2018 was performed via PubMed, Embase, Science Direct, ISI Web of Science as well as three Chinese medical databases: China National Knowledge Infrastructure database (CNKI), VIP database and WanFang database. Risk was assessed by standardized mean difference (SMD) with 95% confidence interval (CI) to investigate the involvement of CRP levels in PD patients.

**Results:** Twenty-three eligible case-control studies involving 4,598 individuals (2,646 PD patients and 1,932 healthy controls) were incorporated into this meta-analysis. Results have indicated significant increase of CRP levels in PD subjects when compared with control groups in serum (SMD = 1.115, 95% CI 0.619–1.61, *P* < 0.001), CSF (SMD = 1.127, 95% CI 0.133–2.120, *P* = 0.026) as well as whole blood (SMD = 1.071, 95% CI 0.715–1.426, *P* < 0.001).

**Conclusions:** This meta-analysis revealed that PD is associated with an increase of CRP levels. CRP might be a risk factor for PD or PD leads to an inflammatory response.

## Introduction

Parkinson's disease (PD) is one of the most complex neuro-degenerative disorders next to Alzheimer's disease. It is characterized by bradykinesia, tremor, rigidity, abnormal postural, and gait (1). PD has been recognized by the selective loss of dopaminergic neurons within the substantia nigra pars compacta, whereas the exact etiology remains elusive (2). Previously, multiple inferences have reviewed the environmental and behavioral factors as the risk of developing PD, such as high milk and dairy consumption, exposure to pesticides and other environmental chemicals, history of melanoma, usage of amphetamine or methamphetamine, and traumatic brain injury, etc (3). However, the last decades, testable hypotheses were build that microglia-mediated neuroinflammation may contribute to the pathogenesis of PD (4–7). Furthermore, epidemiological studies show that anti-inflammatory medications, especially nonsteroidal anti-inflammatory drugs have neuroprotective effects and greatly reduced the risk of PD, which forcefully corroborating the above mentioned hypothesis (8–11).

C-reactive protein (CRP), a kind of acute-phase protein and regulated by pro-inflammatory cytokines, is the most studied bio-marker of systemic inflammation (12). Some researches suggest that elevated levels of CRP is intensely associated with inflammatory process (13). Studies have also demonstrated a link between CRP and chronic inflammatory and neurodegenerative diseases, such as cardiovascular disease, diabetes, stroke, and Alzheimer's disease, as well as PD (14). Up to now, some epidemiological studies have explored CRP levels and PD risk. However, results in the literature regarding CRP levels in PD patients are still contradictory. Some studies found a significantly increase of CRP levels in subjects suffering PD compared with healthy controls (15–17), while some reports did not identify a similar tendency (18, 19). Therefore, we performed a systematic review via an extensive and comprehensive search that focus on serum, plasma, blood and CSF CRP levels in PD patients to clarify the potential risk of CRP levels in PD.

## Materials and Methods

### Search Strategy

This meta-analysis was performed in line with the preferred reporting items for systematic reviews and meta-analyses (PRISMA) statement (20). A systematic review via PubMed, Embase, Science Direct, ISI Web of Science, and three Chinese medical databases: China National Knowledge Infrastructure database, VIP database, and WanFang database was searched since published from inception till October 2018. Search strategies included a combination of the following key words: (“C-reactive protein” OR “C reactive protein” OR CRP) and (“Parkinson Disease” OR “Parkinson's Disease” OR “Parkinsonism” OR PD). Both English and Chinese articles were enrolled. In addition, potential reviews and meta-analyses were examined manually to identify any additional related article that might be suitable for this review.

### Inclusion and Exclusion Criteria

Two investigators (Qiu X, Xiao Y) independently identified and selected studies based on the Participants, Intervention, Comparison, Outcomes, and Study design (PICOS) framework (Table 1). Regarding to the reduplicative patient population, only the latest or most complete study was recruited.

**Table 1:** PICOS of all publications.

**Study**	**Participants**	**Intervention**	**Comparators**	**Outcomes**	**Study design**
Song (21)	Inclusion criteria: *de novo* PD patients without a history of antiparkinsonian drug therapy diagnosed according to the UKPDSBB Clinical Diagnostic Criteria. Exclusion criteria: patients with cognitive impairment, secondary causes of parkinsonism, neuroleptic drug use or psychiatric diseases, cerebrovascular disease or focal neurological signs of cerebral disease.	No intervention required	Healthy control subjects	Serum hs-CRP level	Case control study
Song (22)	Inclusion criteria: consecutive newly diagnosed early stage PD patients with Hoehn and Yahr stage 1 and motor symptoms according to the UKPDSBB Clinical Diagnostic Criteria. Patients should be without a history of antiparkinsonian drug therapy. Exclusion criteria: patients with cognitive impairment, secondary causes of parkinsonism, neuroleptic drug use or psychiatric diseases, cerebrovascular disease or focal neurological signs of cerebral disease with complaints of motor symptoms.	No intervention required	Normal controls were free of any medical abnormality, such as an infection or neurological deficit, and were determined to be free of risk factors of stroke.	Serum hs-CRP level	Case control study
Liu (23)	Inclusion criteria: newly diagnosed PD patients according to the PD and Parkinson's syndrome criterion proposed in the National Extrapyramidal Disease Conference in China 1984. Patients should be without a history of antiparkinsonian drug therapy. Exclusion criteria: NA	No intervention required	Healthy controls without nervous or immunity system disease.	Blood and CSF CRP level	Case control study
Andican (15)	Inclusion criteria: idiopathic PD patients with the treatment with levodopa for at least 6 months. Exclusion criteria: patients with hypothyroidism, coronary heart disease, renal or hepatic insufficiencies, cognitive impairment or recent infectious diseases or having the risk factors for cerebrovascular disease.	No intervention required	Healthy control subjects	Plasma hs-CRP level	Case control study
Qu (24)	Inclusion criteria: idiopathic PD patients diagnosed according to the UKPDSBB Clinical Diagnostic Criteria. Exclusion criteria: patients with other nervous system diseases, recent infectious diseases or usage of non-steroidal anti-inflammatory drugs.	No intervention required	Healthy controls without history of coronary heart disease, cerebral infarction and diabetes disease, connective tissue disease, liver and kidney disease and thrombotic diseases.	Plasma CRP level	Case control study
Song (25)	Inclusion criteria: consecutive PD patients with or without dementia with a clinical dementia rate score of 0.5 and a mini-mental status examination score of 24 points diagnosed according to the UKPDSBB Clinical Diagnostic Criteria and the Diagnostic and Statistical Manual of Mental Disorders, 4th edition text revision criteria for dementia. Exclusion criteria: patients with secondary causes of Parkinsonism.	No intervention required	Healthy controls without history or symptoms of PD, memory impairment or other types of cognitive impairment and other neurological diseases.	Serum hs-CRP level	Case control study
Wang (26)	Inclusion criteria: idiopathic PD patients with non-motor symptoms diagnosed according to the UKPDSBB Clinical Diagnostic Criteria. Exclusion criteria: patients with cerebrovascular disease, encephalitis of cerebrovascular disease, encephalitis and other reasons caused parkinsonism.	No intervention required	Healthy controls without cerebrovascular disease, encephalitis of cerebrovascular disease, encephalitis and other reasons caused parkinsonism.	Serum hs-CRP level	Case control study
Liu (27)	Inclusion criteria: newly diagnosed PD patients according to the Clinical Diagnosis Standard proposed by Neurology branch of Chinese medical association movement disorder and Parkinson's group in China. Exclusion criteria: patients with infectious disease, schizophrenia, secondary dementia, chronic alcoholic poison, cerebrovascular disease, intracranial tumor, head injury, degeneration diseases, depression, anxiety and dementia.	No intervention required	Healthy control subjects	Serum hs-CRP level	Case control study
Song (16)	Inclusion criteria: *de novo* PD patients without a history of antiparkinsonian drug therapy diagnosed according to the UKPDSBB Clinical Diagnostic Criteria. Exclusion criteria: patients with cognitive impairment, secondary causes of parkinsonism, neuroleptic drug use or psychiatric diseases, cerebrovascular disease or focal neurological signs of cerebral disease.	No intervention required	Healthy control subjects	Serum hs-CRP level	Case control study
Gao (28)	Inclusion criteria: newly diagnosed PD patients with non-motor symptoms according to the PD and Parkinson's syndrome criterion proposed in the National Extrapyramidal Disease Conference in China. Exclusion criteria: patients with essential tremor, parkinsonism, Parkinson-Plus syndromes, malignant tumor, other mental illness and usage of nonsteroidal anti-inflammatory drugs.	No intervention required	Healthy control subjects	Serum hs-CRP level	Case control study
Li (29)	Inclusion criteria: sporadic PD patients diagnosed according to the UKPDSBB Clinical Diagnostic Criteria. Exclusion criteria: patients with Parkinson syndrome.	No intervention required	Healthy control subjects without a family history of PD.	Blood CRP level	Case control study
Zhu (30)	Inclusion criteria: PD patients diagnosed according to the PD and Parkinson's syndrome criterion proposed in the National Extrapyramidal Disease Conference in China. Exclusion criteria: patients with parkinsonism, Parkinson-Plus syndromes and latest infection.	No intervention required	Healthy control subjects without latest infection.	Blood hs-CRP level	Case control study
de Farias (17)	Inclusion criteria: PD patients diagnosed according to the UKPDSBB Clinical Diagnostic Criteria. Exclusion criteria: patients with clinical or laboratory evidence of autoimmune, renal, heart, liver and other neurological diseases, alcohol dependence and use of antioxidant supplements	No intervention required	Healthy control subjects without autoimmune, renal, heart, liver and other neurological diseases, alcohol dependence and use of antioxidant supplements.	Blood CRP level	Case control study
Wang (31)	Inclusion criteria: idiopathic PD patients diagnosed according to the diagnosis of Parkinson's disease in 2005. Exclusion criteria: patients with other neurological diseases, autoimmune diseases, severe dementia, malignant tumor, severe dysfunction of the liver and the kidney and receiving non-steroidal anti-inflammatory drugs or glucocorticoids.	No intervention required	Healthy control subjects	Blood hs-CRP level	Case control study
Luan (32)	Inclusion criteria: PD patients diagnosed according to the UKPDSBB Clinical Diagnostic Criteria. Exclusion criteria: patients with renal, heart, liver and other neurological diseases, severe anxiety and depression, malignant tumor, and receiving antibiotic drugs or antipsychotics.	No intervention required	Healthy control subjects	Serum CRP level	Case control study
Han (33)	Inclusion criteria: PD patients diagnosed according to the UKPDSBB Clinical Diagnostic Criteria. Exclusion criteria: patients with parkinsonism, Parkinson-Plus syndromes, severe dementia, malignant tumor, and receiving antibiotic drugs or glucocorticoids.	No intervention required	Healthy control subjects	Serum hs-CRP level	Case control study
Li (34)	Inclusion criteria: PD patients diagnosed according to the UKPDSBB Clinical Diagnostic Criteria. Exclusion criteria: patients with parkinsonism, Parkinson-Plus syndromes, severe dementia, renal, heart, liver diseases, malignant tumor, autoimmune diseases, latest infection and receiving antibiotic drugs or glucocorticoids.	No intervention required	Healthy control subjects without depression.	Serum CRP level	Case control study
Tang (35)	Inclusion criteria: PD patients diagnosed according to the UKPDSBB Clinical Diagnostic Criteria. Exclusion criteria: patients with secondary causes of Parkinsonism, other neurological diseases, tumor, autoimmune diseases and latest infection.	No intervention required	Healthy control subjects without tumor, autoimmune diseases and latest infection.	Plasma hs-CRP level	Case control study
Williams-Gray (18)	Inclusion criteria: idiopathic PD patients diagnosed according to the UKPDSBB Clinical Diagnostic Criteria. Exclusion criteria: NA.	No intervention required	Healthy control subjects	Serum CRP level	Case control study
Starhof (36)	Inclusion criteria: PD patients. Exclusion criteria: NA.	No intervention required	Healthy control subjects	CSF CRP level	Case control study
Baran (37)	Inclusion criteria: PD patients diagnosed according to the UKPDSBB Clinical Diagnostic Criteria. Exclusion criteria: patients with neurological disorder, inflammatory or autoimmune disorder, or an active infection, severe systemic disease, diabetes mellitus, chronic heart disease, liver or kidney failure, alcohol or substance abuse, a history of severe head trauma and myocardial infarction.	No intervention required	Healthy control subjects without neurological or infectious disease.	Serum hs-CRP level	Case control study
Hall (19)	Inclusion criteria: PD patients diagnosed according to the National Institute of Neurological Disease and Stroke Diagnostic Criteria. Exclusion criteria: NA.	No intervention required	Healthy control subjects	CSF CRP level	Case control study
Sanjari Moghadd (38)	Inclusion criteria: PD patients. Exclusion criteria: patients with other neurological diseases.	No intervention required	Healthy control subjects without any neurological diseases.	CSF CRP level	Case control study

*PD, Parkinson's disease; UKPDSBB, United Kingdom Parkinson's Disease Society Brain Bank; CRP, C-reactive protein; SD, standardized difference; CSF, cerebrospinal fluid; NA, not-available*.

### Data Extraction

Data was extracted from enrolled articles independently by two investigators (Qiu X, Xiao Y). We collected the following data: (1) general information: first author, publication year, country of the population and study subjects; (2) patient characteristics: sample size, age, gender, clinical setting, adjusted variables for controls; (3) CRP assay type; (4) CRP level. Inconsistencies between the two authors were resolved by consulting a third reviewer (Wang J), and a level of 95% agreement was achieved. We contacted the investigators of the research for further information when required.

### Quality Assessment

Quality of each study was evaluated on the basis of the Newcastle-Ottawa Scale (NOS) (39). There were three domains outlined in the NOS: “Selection,” “Comparability,” and “Outcome.” NOS scores with nine points were adopted to determine the quality categories. High quality was defined with 7 scores or more, intermediate quality from 4 to 6 and inferior quality below 4.

### Statistical Analysis

The software STATA version 12.0 (StataCorp LP, College Station, TX, USA) was employed to perform this meta-analysis investigating the correlation of PD risk and CRP levels. Standardized mean difference (SMD) with 95% confidence interval (CI) for continuous outcomes was used to measure the differences of CRP levels between PD patients and healthy controls. The value of SMD < 0.2 implied a slight effect, indicating a low risk between CRP levels and PD, 0.5 indicated a moderate effect, and exceeding 0.8 suggested a significant effect (40). Heterogeneity among trials were quantified by *Q* chi-square test and *I*^2^ metric. Substantial heterogeneity exists when *I*^2^ exceeding 50% and *P*-value < 0.5 for the *Q* test (41). A random- effects model was employed to merge the overall effect size if significant heterogeneity existed; otherwise, a fixed-effects model was preferred (42). Subgroup analyses were conducted to explore the possible causes of heterogeneity. Sensitivity analysis was utilized to value the influence of individual research on the overall effect estimate. Funnel plot was applied to visually inspect publication bias, along with Egger's test (43). *P*-values < 0.05 was supposed to statistically significant.

## Results

### Studies Selection

**Figure 1:**
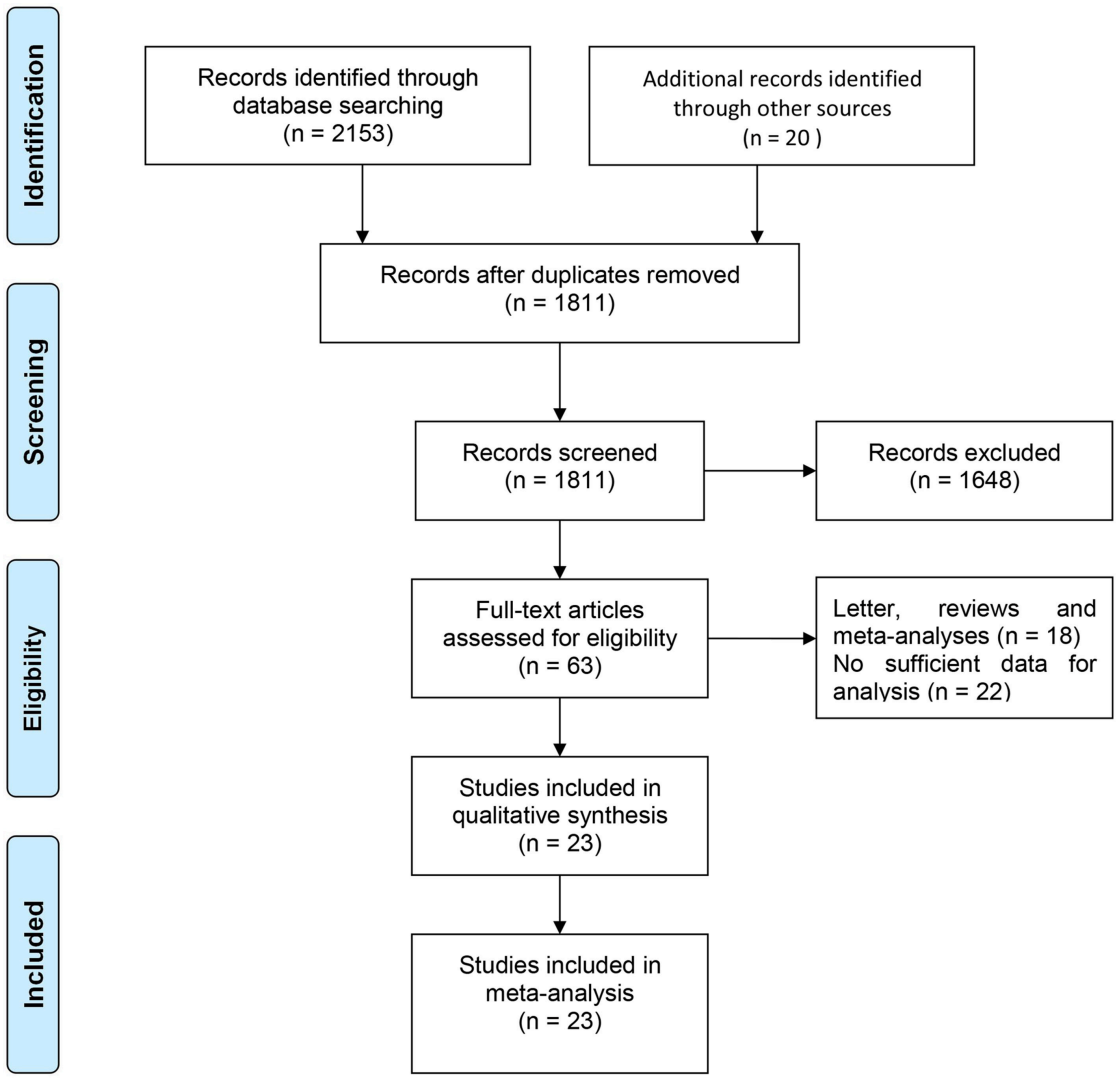
Flowchart of the study identification, inclusion, and exclusion in the meta-analysis.

A flowchart describing the study selection process is displayed in Figure 1. A total of 2,173 citations were yielded based on the search strategies above. After duplicates were removed, the remaining 1,811 articles were then independently reviewed. When filtrating titles and abstracts, a majority of 1,648 papers were excluded given that they were out of the predetermined criteria. Finally, 23 studies fulfilling the inclusion criteria were included in this meta-analysis (15–19, 21–38).

### Study Characteristics

The descriptive data of the 23 studies are outlined in Table 2. The date of publication was between 2009 and 2018. Sample size ranged from 40 to 800 subjects, with a number of 4,589 participants were included (a total of 2,646 cases and 1,932 controls). The mean age of PD patients ranged from 63.6 to 73.2 years. The disease duration of PD varied from 3 months to 9.8 years. Nineteen (15, 16, 21–35, 37, 38) studies recruited participants in Asian, three (18, 19, 36) in Europe and the remaining one (17) in South America. PD patients in nine researches (15, 17, 18, 24, 26, 32, 35–37) were taking anti-PD drugs such as levodopa. Eleven trials (18, 19, 24–29, 31, 33, 34) enrolled the PD patients accompanied by dementia, anxiety or depression. Samples of 12 trials (16, 18, 21, 22, 25–28, 32–34, 37) were from serum, three (15, 24, 35) from plasma, four (17, 29–31) from the whole blood, three (19, 36, 38) from CSF, and one (23) from both blood and CSF. Thirteen studies (15, 16, 21, 22, 25–28, 30, 31, 33, 35, 37) applied high-sensitivity CRP (hs-CRP) assay and 10 (17–19, 23, 24, 29, 32, 34, 36, 38) utilized standard CRP maker. The quality of the enrolled studies was assessed by means of Newcastle-Ottawa Scale. Ten (16, 17, 19, 21, 22, 24, 25, 29, 37, 38) out of 23 studies were assessed of high quality, 11 studies (18, 23, 26–28, 30, 31, 33–36) were assessed as medium quality, and the remaining two studies (15, 32) were evaluated as low quality.

**Table 2:** Characteristics of the studies included in this meta-analysis.

**Author [References]**	**Year**	**Country**	**No. (P/C)**	**Disease duration mean ± SD or range**	**Age (P/C) mean ± SD (y)**	**Adjustments**	**Anti-PD drug**	**Comorbidity**	**CRP assay type**	**NOS score**
Song (21)	2009	Korea	212/119	20.72 ± 17.97 (m)	68.74 ± 9.32/66.59 ± 12.63	Age; gender	No	No	NA	8
Song (22)	2011	Korea	63/117	6.03 ± 6.75 (m)	63.68 ± 10.16/66.57 ± 12.70	Age; gender	No	No	NA	8
Liu (23)	2012	China	20/20	9.8 ± 2.9 (y)	62.2 ± 5.6/60.2 ± 8.2	NA	No	No	Endpoint nephelometry	6
Andican (15)	2012	Turkey	45/25	6.4 ± 4.3 (y)	63.6 ± 13.5/60.2 ± 12.5	Age	Yes	No	ELISA	4
Qu (24)	2012	China	40/40	3.8 ± 2.9 (y)	68 ± 10/60 ± 10	Age; gender	Yes	Anxiety; depression	Emulsion immunoturbidimetric	7
Song (25)	2013	Korea	117/84	21 ± 18.65 (m)	70.4 ± 11.6/73.18 ± 19.75	Age; gender	NA	Dementia	NA	8
Wang (26)	2014	China	50/30	3 m−12 y	66 ± 8.5/NA	NA	Yes	Anxiety; depression	Immune turbidimetric test	5
Liu (27)	2014	China	89/46	5.64 ± 3.81 (y)	70.8 ± 5.18/69.7 ± 5.3	Age; gender; education years	NA	Cognitive impairment	Enzymatic colorimetry	6
Song (16)	2014	Korea	435/221	39.49 ± 33.99 (m)	69.39 ± 9.19/68.47 ± 11.48	Age; gender	No	No	NA	7
Gao (28)	2015	China	80/80	3.7 ± 2.4 (y)	66.3 ± 7.5/64.7 ± 6.9	Age; gender; education years	NA	Anxiety; depression	NA	6
Li (29)	2015	China	400/400	NA	62.6 ± 11.02/63.22 ± 10.61	Age; gender	NA	Anxiety; depression	NA	7
Zhu (30)	2016	China	52/50	NA	64.32 ± 2.14/64.02 ± 3.41	Age; gender	NA	No	NA	6
de Farias (17)	2016	Brazil	56/56	6.5 ± 4.2 (y)	70.3 ± 8.9/69.7 ± 8.8	Age; gender; ethnicity	Yes	No	Automated immunological method	7
Wang (31)	2016	China	62/62	NA	65.02 ± 7.21/64.61 ± 7.51	Age; gender	NA	Anxiety; depression	ELISA	5
Luan (32)	2016	China	102/42	6.45 ± 1.21 (y)	66.99 ± 10.82/68.41 ± 11.23	NA	Yes	No	ELISA	4
Han (33)	2016	China	83/80	5.1 ± 0.2 (y)	54.5 ± 4.3/53.2 ± 5.6	Age; gender	NA	Anxiety; depression	Immunotransmission turbidimetric method	6
Li (34)	2016	China	113/52	3 ± 1.06 (y)	65.15 ± 7.15/NA	Age; gender	NA	Depression	ELISA	6
Tang (35)	2016	China	126/120	NA	63.68 ± 15.16/61.56 ± 18.7	Age; gender	Yes	No	Immunotransmission turbidimetric method	6
Williams-Gray (18)	2016	UK	230/93	0.6 ± 0.5 (y)	66.4 ± 9.5/68.0 ± 8	Age	Yes	Dementia	Electrochemiluminesc-ent immunoassays	6
Starhof (36)	2018	Denmark	46/31	83.78 ± 45.9 (m)	64.46 ± 11.5/45.48 ± 17.7	NA	Yes	No	NA	6
Baran (37)	2018	Turkey	30/30	5.9 ± 4.7 (y)	70.7 ± 9.6/66.8 ± 9.0	Gender; age; body mass index	Yes	No	ELISA	7
Hall (19)	2018	Sweden	131/50	5.5 ± 4.8 (y)	64.9 ± 10.6/65.3 ± 8.6	NA	NA	Dementia	ELISA	7
Sanjari Moghadd (38)	2018	Iran	109/84	104.93 ± 40.47 (m)	69.71± 6.55/67.1 ± 7.2	Education years	NA	No	NA	8

*No., number of participants; P, patients; C, controls; SD, standardized difference; PD, Parkinson's disease; NOS, Newcastle-Ottawa Scale; NA, not available; y, years; m, months; ELISA, enzyme-linked immunosorbent assay*.

### Meta-Analysis of CRP in Blood Levels

**Figure 2:**
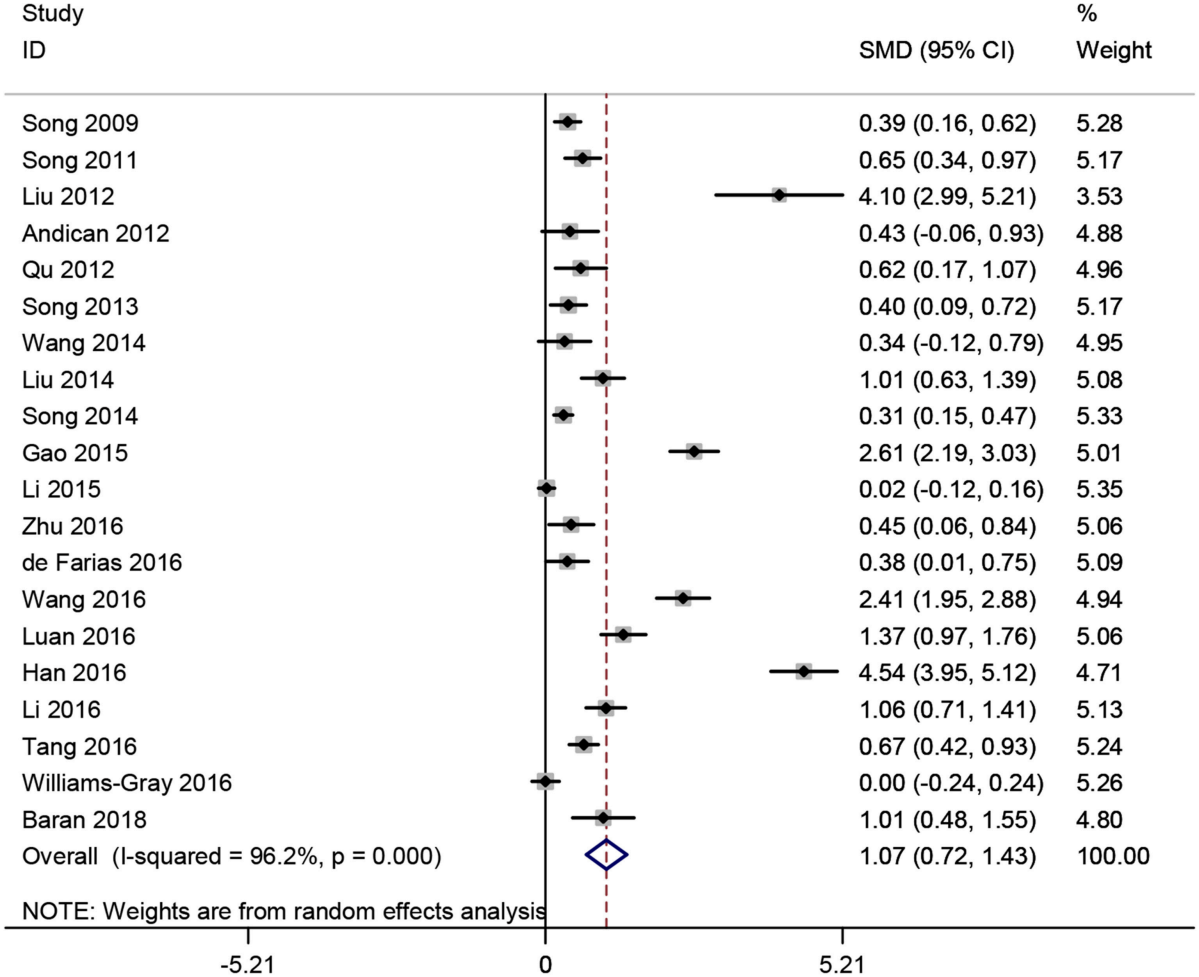
Forest plot of blood C-reactive protein (CRP) levels between Parkinson's disease (PD) patients and healthy controls. The size of square size reflects the study's weight. Each horizontal line represents the 95% confidence interval of standardized mean difference. Diamond represents the pooled standardized mean difference. SMD, standardized mean difference; CI, confidence interval.

Twenty studies (15–18, 21–35, 37) measured CRP levels in peripheral blood consisting of those samples derived from serum, plasma or whole blood of PD patients. A total of 4,127 subjects with 2,360 PD patients and 1,767 normal controls were included in this meta-analysis. Results showed that CRP levels in peripheral blood were significantly increased in PD patients compared to controls (SMD = 1.071, 95% CI: 0.715–1.426, *P* < 0.001) (Figure 2), a random-effect model was used because of the heterogeneity (*I*^2^ = 96.2%, *P* < 0.001).

**Figure 3:**
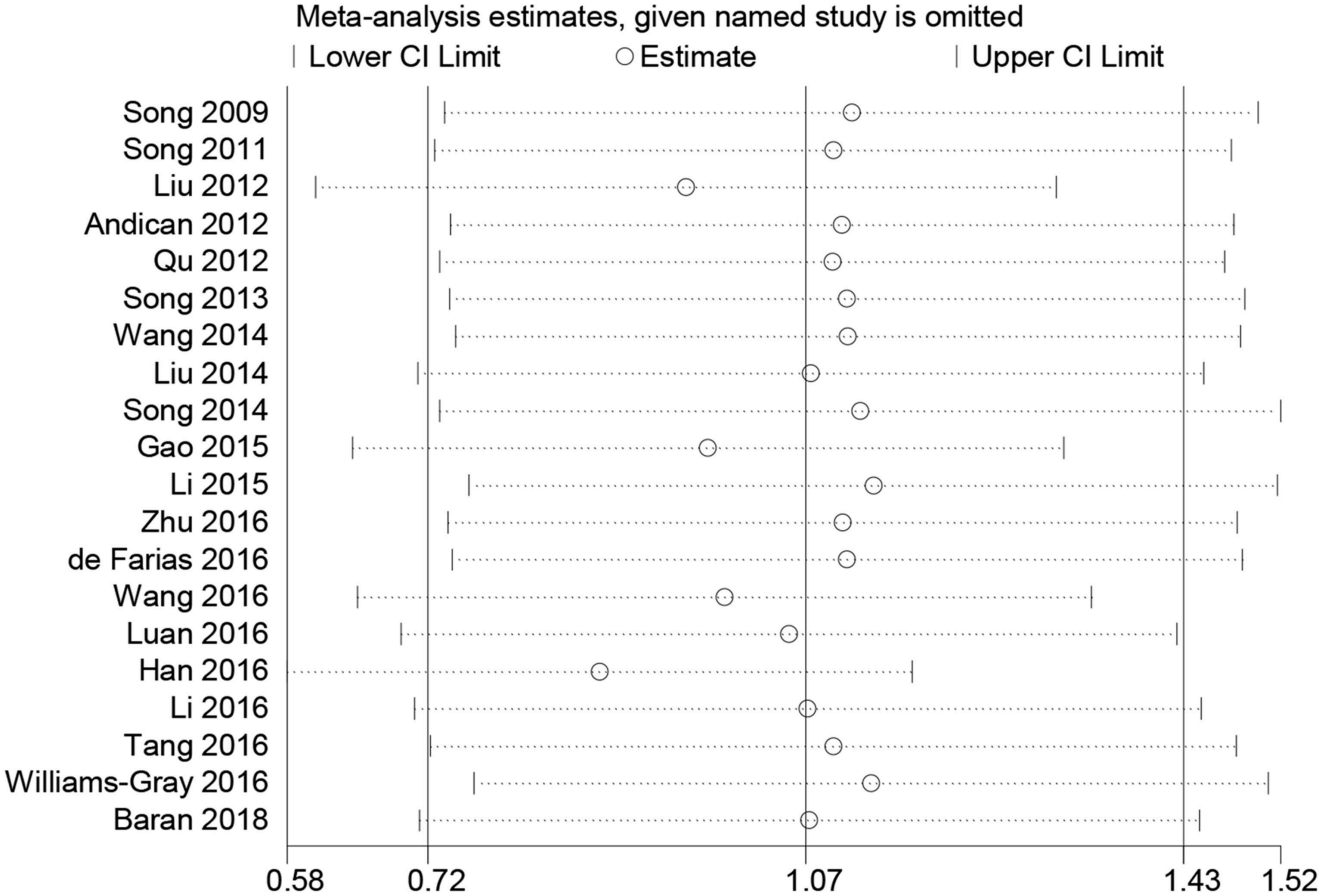
Sensitive analysis indicated the robustness of results. CI, confidence interval.

**Figure 4:**
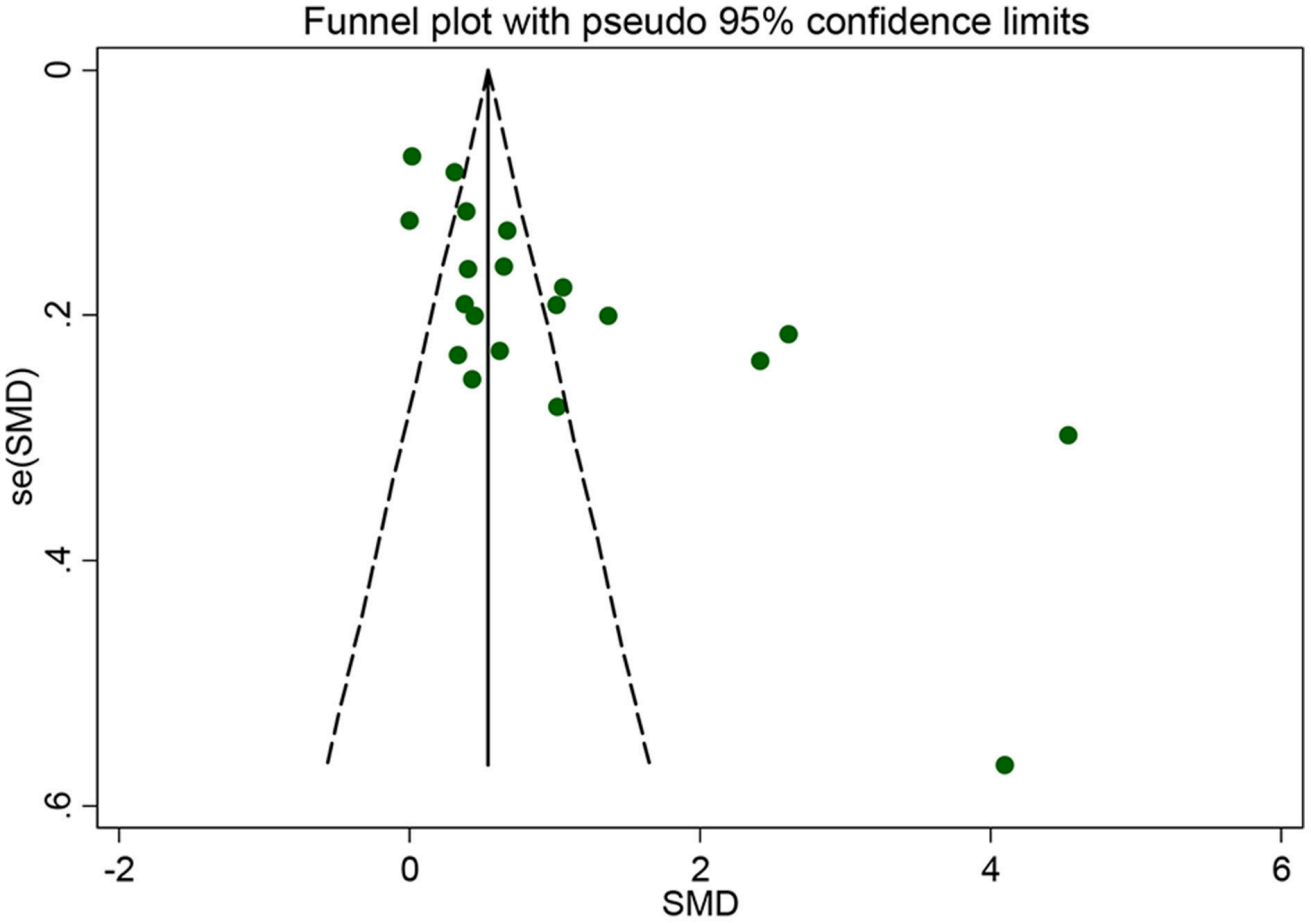
Funnel plot of the selected studies. It suggests publication bias. SMD, standardized mean difference.

Subgroup analyses were conducted according to CRP measurement manner. Results suggested that CRP levels were significantly increased in studies using hs-CRP as a measurement method (SMD = 1.150, 95% CI: 0.655–1.634, *I*^2^ = 96.5%). Sensitivity analysis was conducted to address the potential heterogeneity on account of the impact of enrolled studies on the overall effect size and its quality (Figure 3). The pooled SMD in CRP levels ranged from 0.877 (95% CI: 0.583–1.171) to 1.134 (95% CI: 0.755–1.514). Summary results were not influenced significantly by any one particular study, indicating strength and robustness of this meta-analysis. Asymmetry was identified in the funnel plot where four studies (23, 28, 31, 33) fell distantly outside of the predicted 95% CI, implying a potential publication bias should not be ignored (Figure 4). Egger's test (*P* < 0.001) also provided statistical evidence of publication bias.

### Meta-Analysis of CRP in Serum Levels

**Figure 5:**
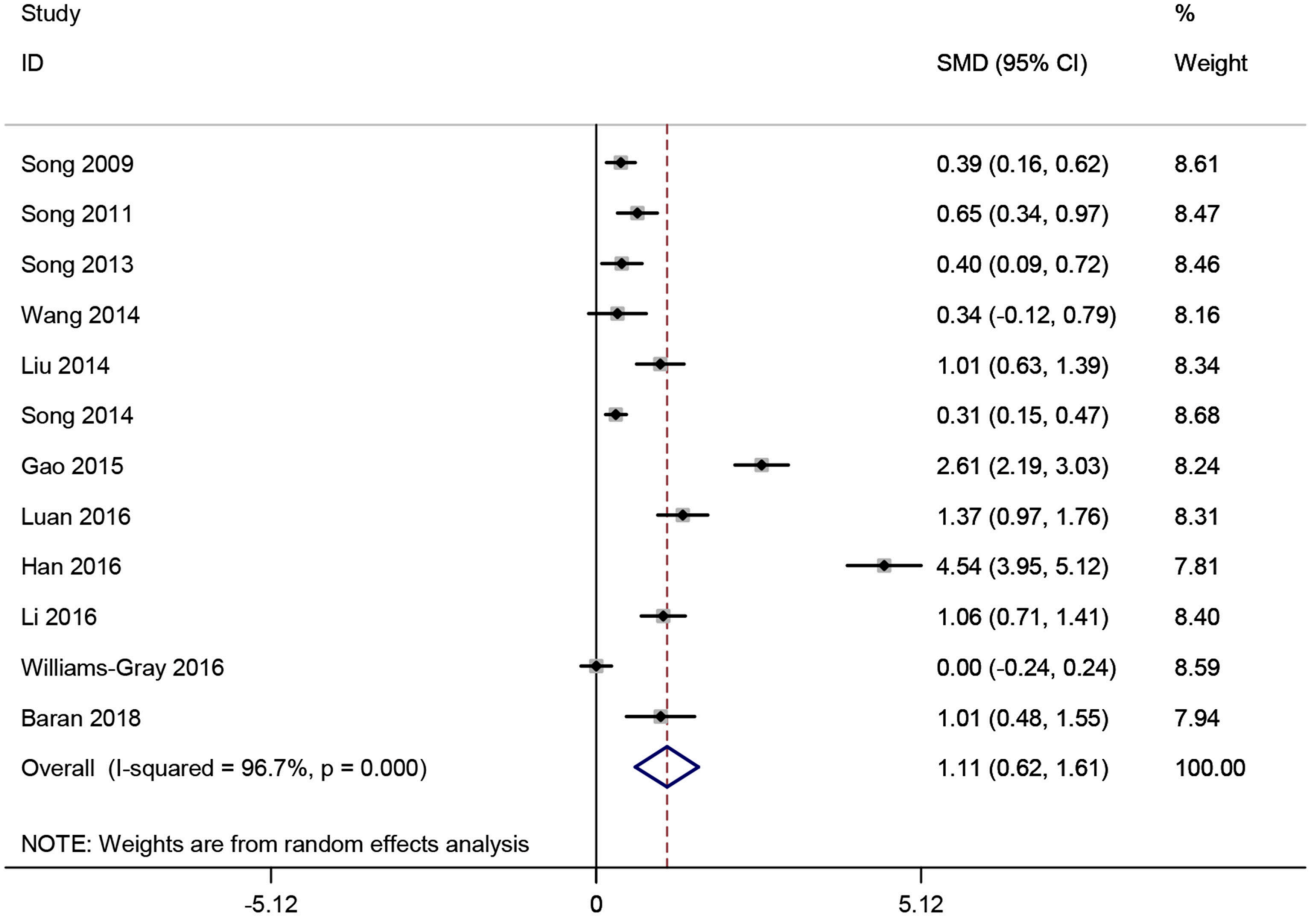
Forest plot of serum C-reactive protein (CRP) levels between Parkinson's disease (PD) patients and healthy controls. The size of square size reflects the study's weight. Each horizontal line represents the 95% confidence interval of standardized mean difference. Diamond represents the pooled standardized mean difference. SMD, standardized mean difference; CI, confidence interval.

Twelve studies (16, 18, 21, 22, 25–28, 32–34, 37) involving 2,553 participants that comparing the serum CRP levels in PD patients and healthy controls were analyzed. Nine studies (16, 21, 22, 25–28, 33, 37) employed the hs-CRP maker to measure the serum CRP levels while the remaining three (18, 32, 34) used CRP. In this meta-analysis, the serum CRP levels were significantly higher in PD patients compared to those healthy controls (SMD = 1.115, 95% CI: 0.619–1.61, *P* < 0.001, Figure 5). Due to the substantial heterogeneity detected (*I*^2^ = 96.7%, *P* < 0.001), a random-effect model was employed (44, 45). Because of the similar clinical characteristics among these 12 studies, we did not carry out subgroup analyses. No individual study remarkably influenced the stability of the results in sensitivity analysis. However, Egger's linear regression test (*P* = 0.01) found the exist of publication bias.

### Meta-Analysis of CRP in CSF Levels

**Figure 6:**
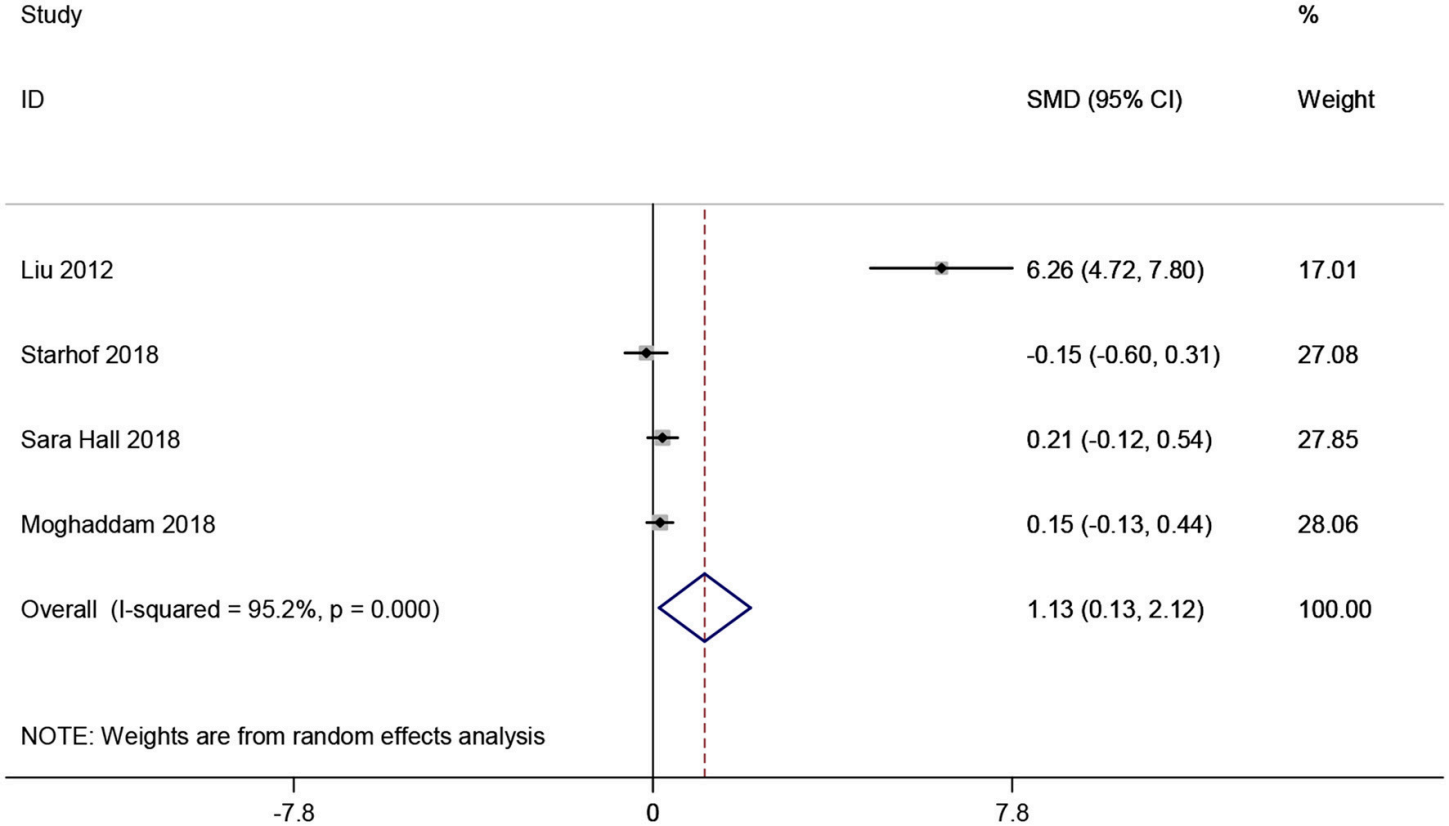
Forest plot of cerebrospinal fluid (CSF) C-reactive protein (CRP) levels between Parkinson's disease (PD) patients and healthy controls. The size of square size reflects the study's weight. Each horizontal line represents the 95% confidence interval of standardized mean difference. Diamond represents the pooled standardized mean difference. SMD, standardized mean difference; CI, confidence interval.

CRP levels in CSF were analyzed in 491 participants from four studies (19, 23, 36, 38). Pooled analysis revealed that CRP levels in CSF were greatly increased in studies with PD patients compared to healthy controls (SMD = 1.127, 95% CI: 0.133–2.120, *P* < 0.001, Figure 6). Since obvious heterogeneity among those studies (*I*^2^ = 95.2%, *P* < 0.001) were observed, a random-effect model was utilized. We did not perform further subgroup analysis considering the limited number of included studies.

## Discussion

In recent years, the association between CRP and risk of PD has been widely studied. However, it is still not well understood. Herein, in this meta-analysis, we made a comprehensive comparison of the CRP levels in peripheral blood and CSF between PD patients and healthy controls. A total of 23 eligible case-control studies recruiting 4,598 participants were enrolled. Our results suggested that elevated CRP levels in both the serum and CSF were prominently in PD patients. Besides, an unite analysis for peripheral blood and CSF strengthens the outcomes. Subgroup analyses displayed that irrespective of CRP or hs-CRP being the measurement maker, the CRP levels in peripheral blood significantly increased in PD patients comparing with the controls. In addition, sensitivity analysis appeared to indicate that the results were stable. Nevertheless, funnel plot with slight asymmetry and Egger's test (*P* < 0.001) did detect the occurrence of publication bias because of the four studies (23, 28, 31, 33). Study by Liu et al. (23) recruited the smallest sample size of only 40 participants and PD patients with the longest duration. The patients and controls enrolled in research by Han et al. (33) were the youngest groups with age no more than 55 years old. Those two studies both reported extremely large effect, and results of Gao et al. (28) and Wang et al. (31) also exerted quite an influence on overall effect size. Notwithstanding, those four studies did not change the general results in sensitivity analysis; the publication bias may be due to the unpublished negative studies and the existence of the high heterogeneity among the studies. In all, the results indicated an association of higher CRP levels and the risk of PD. However, further large scale and well-design trials are warranted to verify our conclusion.

The results of this review only suggested a correlation between CRP levels and PD, but could not completely delineate whether inflammation plays a causal role in PD, or if PD leads to inflammatory processes. Furthermore, some other confounding factors might influence CRP levels in PD patients. There was considerable variation in the data of disease duration in the included studies, suggesting the probable existence of some confounders (e.g., anti-PD drugs, comorbidity). Some studies (15, 35) measured the difference of hs-CRP level in blood that was not statistically significant between PD patients and controls, when the patients were treated with drugs like levodopa, dopamine receptor agonists and so on. Besides, Andican et al. (15) also found no correlation between the hs-CRP level in plasma and the daily dosage of levodopa or the duration of PD. In addition, de Farias et al. (17) recommended that the anti-PD drugs might increase the inflammatory state. Due to the rarely relative studies included, we could not do a further analysis of the confounding factors such as anti-PD drugs and comorbidity which might be had some affects to our conclusion. Moreover, in a cross-sectional study (46), the authors did not find any correlation between plasma CRP levels and disease duration, levodopa dose, depression, psychosis, dementia, or cognitive decline. However, Lindqvist et al. (47) reported a strong correlation between high CRP levels in CSF and disease duration, fatigue and depression and dementia. Thus, further studies are needed to verify an association between confounding factors such as levodopa treatment or comorbidity in PD and CRP levels.

In present, the mechanisms of elevated CRP levels and neuroinflammation underlying the pathophysiology of PD are still not completely elucidated. As a neurodegenerative disorder, PD is associated with progressive dopaminergic neuronal degeneration in the substantia nigra. And its prominent neuropathological feature is the presence of Lewy bodies predominantly composed of fibrillar α-synuclein (1). At the cellular level, aggregated α-synuclein can promote microglial activation and stimulate the secretion of inflammatory molecules, evoking neuroinflammation. In turn, neuroinflammation may trigger cascade of deleterious events, such as oxidative stress and cytokine-receptor-mediated apoptosis, thereby exacerbating dopaminergic neurodegeneration (5, 48). Furthermore, epidemiological studies observed that long-term taking anti-inflammatory medications could delay or prevent dopaminergic cell death through inhibiting the pro-inflammatory responses of microglia (11). Simultaneously, as one the most important bio-maker of inflammation, CRP could also be generated by neurons and microglia in the central nervous system according to the post-mortem studies on patients with AD or intracerebral hemorrhage, or animal studies (49–51). Taken together, there is growing evidence that support an association between neuroinflammation and the initiation and progression of PD pathophysiology.

As we know, this is the most comprehensive meta-analysis of all the published studies that focused on the CRP levels in peripheral blood and CSF between PD patients and matched controls. Though a previous review conducted by Qin (52) assessed several peripheral inflammatory cytokine levels in PD, they only included data from six studies for analyzing CRP levels in PD patients. In this review, a total of 4,598 participants from 23 studies were recruited to estimate the risk, which largely enhanced the reliability of the results. In addition, we focused on CRP levels not only in peripheral blood, but also CSF, which might reflect inflammatory process both of periphery and central nervous system. Our results indicated a significant higher CRP levels in both blood and CSF in patients suffering PD compared with controls. What's more, most of the included studies were of high quality, which further supported that inflammatory cytokine CRP might be involved in the pathological mechanism of PD.

There are still some limitations that should be carefully interpreted. Firstly, though we performed subgroup analyses to reduce the source of heterogeneity, there were remaining substantial heterogeneity, implying that some confounding variables might exit. Secondly, some studies were not included in this meta-analysis as they assessed the median absolute values of CRP in PD patients and healthy controls and data from these studies were unable to extract for analyses (53–56). Thirdly, the exist of publication bias may also affect the results of this review.

## Conclusion

In summary, the current systematic review reveals the CRP levels of blood and CSF were significantly elevated in PD patients when compared with healthy controls, indicating that CRP might be a risk factor for PD or PD leads to an inflammatory response.

## Author Contributions

XQ and YX designed the study, reviewed the literature, conducted the statistical analysis and drafted of the manuscript collectively. JWu, LG, and YH performed summary tables, edited pictures, and discussed on the manuscript. JWa contributed significantly to the study design and critically revised the final manuscript.

### Conflict of Interest Statement

The authors declare that the research was conducted in the absence of any commercial or financial relationships that could be construed as a potential conflict of interest.
